# Incidence and mortality by pulmonary tuberculosis in Brazil: Trends and projections, 2002-2034

**DOI:** 10.1016/j.ijregi.2024.100514

**Published:** 2024-12-06

**Authors:** Jefferson Felipe Calazans Batista, Vitória Steffany de Oliveira Santos, Marcos Antonio Almeida-Santos, Sonia Oliveira Lima

**Affiliations:** Tiradentes University, Health and Environment Post-graduation Program, Aracaju, Sergipe, Brazil

**Keywords:** Pulmonary tuberculosis, Incidence, Mortality, Projections

## Abstract

**Objectives:**

To analyze the temporal trend of incidence and mortality from pulmonary tuberculosis in Brazil from 2002 to 2019 and to project these trends until 2034.

**Methods:**

Ecological study with tuberculosis cases extracted from the Disease Notification and Mortality System in Brazil from 2002 to 2019. The age-period-cohort model was used for projection until 2034 using R. Subsequently, the percentage variation was estimated using Joinpoint.

**Results:**

Brazil recorded 1,093,070 new cases and 76,205 deaths from 2002 to 2019, and projections until 2035-2034 estimated 1,192,092 new cases and 67,532 deaths. The north region had the highest standardized incidence and mortality rates in the country for both sexes. An increase in deaths in men and reduction in women was projected, along with an increase in incidence in both sexes. About 36% of the increase in incidence and 34.1% of the mortality in men was explained by a rise in disease risk. In women, 11.7% of the increase in incidence was due to population growth, whereas 44.8% of the reduction in deaths was due to lower risk.

**Conclusions:**

The north presented the worst scenario in the country. The projections are not favorable to the globally established targets. An increase in incidence was projected for men and women, with an increase in deaths only in men. More efforts are needed to change this potential scenario.

## Highlights

• The north region presented the worst scenario in the country.
• High adjusted incidence and mortality rates were observed in men.
• In general, the trends show decreased incidence and mortality in Brazil by 2034.
• In females, there was a reduction in the risk of dying of tuberculosis.
• It was projected an increase in death risk in the north, south, and center-west regions.

## Graphical Abstract

**Figure:**
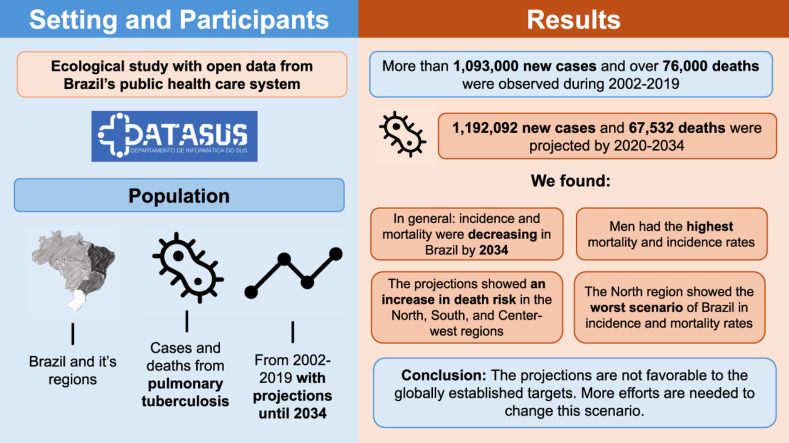

## Introduction

Tuberculosis (TB) has been a global public health challenge marked by significant efforts to reduce its incidence and mortality. The global “Stop TB” strategy, implemented between 1990 and 2015, successfully reduced the disease's prevalence by 42% and deaths by 47%, thanks to increased investments and expanded access to diagnosis and treatment [1].

Despite these advancements, TB remains the leading infectious killer worldwide and the primary cause of death in people living with HIV, surpassing AIDS as the most lethal infectious disease today. Addressing the TB crisis requires a multifaceted approach that encompasses everything from epidemiologic surveillance and rapid diagnostics to effective treatments and preventive measures, such as vaccination and latent TB treatment. The World Health Organization (WHO), through the End TB Strategy, proposes a significant reduction in incidence (10 cases per 100,000) and mortality (1 death per 100,000), aiming to eliminate TB as a public health problem. This goal can only be achieved through continuous innovation, substantial investment, and firm political commitment, highlighting the importance of this study in addressing a persistent and complex issue [2].

The analysis of temporal trends with forecasts for health-related issues is crucial for identifying patterns and determinants that can influence the effectiveness of public health policies, as well as for better guiding control actions, especially in a country such as Brazil, with significant regional disparities. In addition, predicting the future behavior of two important epidemiologic indicators of TB is of great value, given the existing targets for the next 10-15 years, such as the Ministry of Health's goal to eradicate TB by 2035 [3] and the Sustainable Development Goals (2030), which aim to eradicate various diseases, including TB [4].

Therefore, given the relevance of this research and the absence of similar studies in Brazil, the aim was to analyze the distribution and temporal trends of pulmonary TB incidence and mortality in Brazil and its macroregions from 2002 to 2019 and to project these trends through 2034, as well as to determine how variations in disease risk and changes in population size affected these projections.

## Methods

### Study design

A descriptive, exploratory, analytical ecological study with a quantitative approach was conducted following the guidelines of the Reporting of studies Conducted using Observational Routinely-collected health Data [5].

### Setting

Public data related to pulmonary TB in Brazil and its macroregions from January 2002 to December 2019 were used. Brazil has a comprehensive health data system managed by the Department of Informatics of the Unified Health System (*Departamento de Informática do Sistema Único de Saúde*, DATASUS). This department is responsible for collecting, processing, and disseminating public health information, providing essential data for health planning, management, and research in the country. All data can be accessed in https://datasus.saude.gov.br/transferencia-de-arquivos/.

### Participants

All confirmed cases of TB, according to the TB compulsory notification form, and all deaths recorded by the death certificate in the country were considered. Only new cases classified as “new case,” “unknown,” and “post-mortem” in the “entry type” variable were included, as well as deaths according to the International Classification of Diseases codes A15 and A16, corresponding to pulmonary TB with and without bacteriologic and histologic confirmation, respectively.

### Variables

The following variables were selected: detailed age (0->80 years), macroregion of residence (north, northeast, southeast, south, and center-west), sex (male and female), year of diagnosis, and year of death (2002-2019). Data that were marked as “unknown” were not considered due to the impossibility of including them in the data analysis.

Incidence and mortality rates were calculated using the following formula:

$$\frac{o_{i}}{p_{i}}\times 100,000$$

Wherein, o_i_ represents new cases or deaths from pulmonary TB in each location and period and p_i_ represents the resident population in the same location and period. The rates were standardized using the direct method and the WHO's standard population for 2000-2025 [6], also expressed per 100,000 inhabitants. Given that the WHO population by age group starts at 0-4 years, it was necessary to create the groups <1 year and 1-4 years based on the summation by isolated age.

### Data sources

The data source for TB cases was the Information System for Notifiable Diseases, which aggregates information on diseases and conditions of mandatory notification, whereas the mortality data were extracted from the Mortality Information System (SIM), responsible for all mortality data in Brazil. In addition, population estimates were obtained from the Brazilian Institute of Geography and Statistics from the 2010 and 2022 censuses [7,8], inter-census estimates, and projections from 2002 to 2034 [9]. The data extraction took place in April 2024 via Windows Tabulator (TabWin), a free software from the Unified Health System of Brazil, as follows:

Access to the annual databases (2002-2019) of TB notification forms and death certificates on the *Departamento de Informática do Sistema Único de Saúde* (Datasus) website data in *.dbc* format:

1) Importation of the data into Tabwin for conversion to .dbf format,
2) Access to the tabulation files and importation of the converted databases,
3) Tabulation of the data by year and sociodemographic characteristics,
4) Data processing in Microsoft Excel, and
5) Data analysis in R.

### Quantitative variables

The variable “detailed age” was aggregated into 5-year intervals to meet the minimum requirements of the projection technique, as follows: <1, 1-4, 5-9, 10-14, 15-19, 20-25, 26-29, 30-34, 35-39, 40-44, 45-49, 50-54, 55-59, 60-64, 65-69, 70-74, 75-79, and >80 years. The data related to the resident population considered the same 18 age groups. In addition, cases, deaths, and resident population were aggregated into 3-year periods as follows: observed period: 2002-2004 to 2017-2019 and projected period: 2020-2022 to 2032-2034.

### Bias

The years 2020-2022 were affected by the COVID-19 pandemic. The quality of TB notification and death registration was altered during this period [10]. This impacted on the quality of the projections; therefore, data up to 2019 were used. A potential bias of this study is the underreporting of pulmonary TB outcomes in the Information System for Notifiable Diseases, which may not reflect the actual mortality from the disease. To minimize this limitation, data from SIM, which are of higher quality and completeness, were used. The use of SIM data allows a more accurate estimation of TB mortality, reducing the impact of underreporting and providing greater reliability to the results.

### Statistical methods

The projection of incidence and mortality was carried out using the age-period-cohort method through the NORDPRED statistical package (Cancer Registry of Norway, Oslo, Norway), which is available for the R software. This model is considered useful for modeling incidence and mortality events. By simultaneously considering the effects of age, period, and cohort, the projections are more robust and reliable [11]. This is particularly relevant for TB, which is influenced by demographic and temporal factors and widely applied in this field [12,13]. The 3-year intervals were proposed [14], with projections up to 2034, using all six observed periods as the basis for projection. It was assessed whether the changes in projections were attributable to alterations in population size and/or changes in disease risk. This assessment compared the last observed period (2017-2019), with the last projected period (2032-2034) using the following formula:

Δtot=Δrisk+Δpop

=(Nfff−Nooo)

=(Nfff−Noff)+(Noff−Nooo)

Where Δtot = total variation, Δrisk = variation caused by changes in TB death risk, Δpop = variation caused by changes in age groups and population size, Nfff = number of predicted cases for the last projected period, Nooo = number of cases observed in the last observed period, Noff = expected number of cases in the last projected period, with application of the rates from the last observed period, and Nfff − Nooo = annual modification in the number of cases.

The results are expressed as “N,” representing the difference in the number of cases/deaths between the last observed period (2017-2019) and the last projected period (2032-2034); “change,” referring to the difference between the number of projected cases/deaths and the number of expected cases/deaths if the projected population (2032-2034) had maintained the same population size as the last observed period (2017-2019); “risk,” representing, in percentage terms, how much of the change is related to the increase or decrease in disease risk; and “population,” related to how much of the difference in the number of cases/deaths occurred due to changes in population size between the two periods considered [15].

After the projections, temporal trend analyses were conducted using the Joinpoint regression model. Standardized incidence and mortality rates were used for all periods (observed and projected), separated by the country's macroregion and sex. The following parameters were adopted: (i) logarithmic transformation of the dependent variable {ln(y) = xb}; (ii) correction for first-order autocorrelation; (iii) model with homogeneous variance; and (iv) empirical quantile confidence interval [16]*.*

The percentage variation is interpreted as follows:

• Growth trend: Positive 3-year percent change (TPC) and statistically significant model (*P* <0.05)
• Reduction trend: negative TPC and statistically significant model (*P* <0.05)
• Stationary trend: non-significant model (*P* >0.05)

### Ethical considerations

The data used in the present study are open access, freely available, and do not contain any personal identification of individuals, which exempts the need for approval by the research ethics committee.

## Results

In the observed period (2002-2019), Brazil reported 1,093,070 new cases of pulmonary TB, with 735,206 (67.3%) cases in males and 357,864 (32.7%) cases in females. The projections (2020-2034) estimated the occurrence of 878,311 new cases in men and 313,781 in women. Regarding mortality, there were 76,205 total occurrences in the observed period, with 56,926 (74.7%) in men and 19,279 (25.3%) in women, whereas the projections estimated 53,680 deaths in men and 13,853 in women.

**Figure 1:**
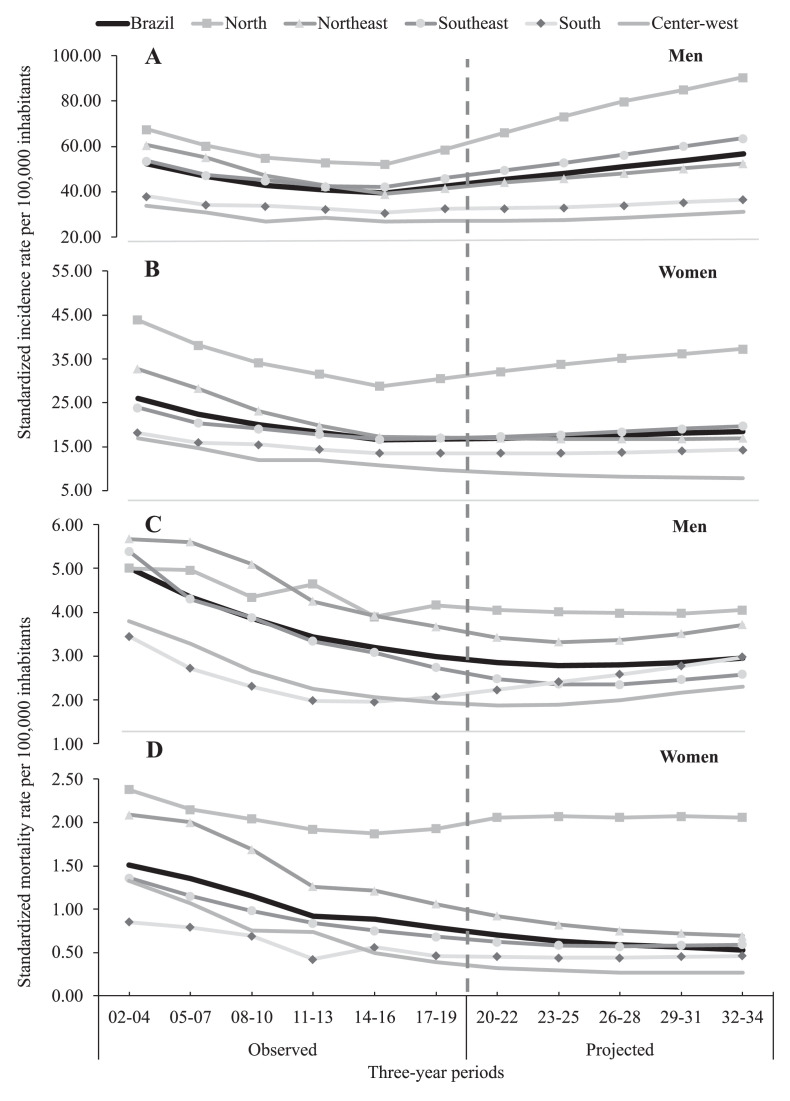
Temporal distribution of pulmonary tuberculosis incidence and mortality by health macroregion and by sex (male and female) in Brazil and macroregions across all periods from 2002-2004 to 2032-2033.

Figure 1 presents the temporal distribution of standardized incidence and mortality rates for pulmonary TB in the observed and projected periods, according to sex and macroregion. Visually, an increase in incidence is observed in both sexes exclusively in the north region (Figure 1a and b). On the other hand, a different scenario is observed for mortality, with a decreasing in all regions, except the north for women (Figure 1c and d).

Table 1 shows the number of new cases and crude and standardized incidence rates separated by sex. In men, high standardized rates were observed in the north and southeast and lower rates in the center-west. In women, there was a predominance in the north and northeast, with the center-west showing the lowest rates. In addition, the north region was the only one to show a high rate in both sexes.

**Table 1:** Number of cases and crude and standardized incidence rates per 100,000 inhabitants by sex in Brazil and macroregions in the observed (2002-2019) and projected (2020-2034) periods.

Region	Observed							Projected			
	02-04	05-07	08-10	11-13	14-16	17-19	20-22	23-25	26-28	29-31	32-34
**Men**											

**North**											
0-19	1,467	1,225	1,153	1,219	1,284	1,490	1,640	2,037	2,152	2,181	2231
20-39	4,718	4,621	4,798	5,220	5,808	7,487	8,853	10,007	11,205	11,971	12,938
40-59	3,055	3,177	3,323	3,582	3,654	4,157	5,257	6,419	7,814	9,297	10,519
60 years+	1,598	1,625	1,647	1,795	1,889	2,240	2,732	3,165	3,592	4,016	4573
Total	10,838	10,648	10,921	11,816	12,635	15,374	18,483	21,627	24,762	27,465	30,261
CR/100 thousand	51.74	46.68	46.61	46.67	47.92	56.05	64.89	73.49	81.75	88.35	95.14
SR/100 thousand	67.61	60.31	54.91	52.99	52.08	58.54	66.05	73.15	79.91	85.08	90.51
**Northeast**											
0-19	3,522	3,156	2,566	2,484	2,299	2,384	2,181	2,423	2,772	2,721	2721
20-39	15,183	14,603	14,015	13,703	13,450	15,862	17,318	17,825	17,621	17,394	17,946
40-59	11,869	11,844	11,741	11,285	10,531	11,091	12,633	14,215	16,466	19,031	20,539
60 years+	5,657	5,562	5,281	5,262	5,165	5,693	6,316	6,895	7,398	7,840	8469
Total	36,231	35,165	33,603	32,734	31,445	35,030	38,448	41,358	44,256	46,985	49,675
CR/100 thousand	49.90	46.31	42.93	40.79	38.60	42.39	45.88	48.76	51.64	54.39	57.18
SR/100 thousand	60.63	55.08	47.15	42.62	39.04	41.59	44.11	46.07	48.15	50.24	52.45
**Southeast**											
0-19	4,200	3,845	3,887	4,091	3,868	4,576	4,172	4,422	4,776	4,856	4902
20-39	23,832	23,643	25,062	25,111	26,914	31,760	36,059	38,358	39,192	39,149	39,688
40-59	21,514	19,889	19,529	18,820	18,122	18,292	20,647	23,461	27,762	33,042	37,352
60 years+	6,754	6,376	6,278	6,548	7,492	8,191	8,315	8,701	9,115	9,746	10,641
Total	56,300	53,753	54,756	54,570	56,396	62,819	69,193	74,942	80,844	86,793	92,584
CR/100 thousand	50.87	46.07	46.56	44.72	45.07	49.02	52.82	56.11	59.53	63.03	66.50
SR/100 thousand	53.57	47.31	44.96	42.41	42.26	45.95	49.46	52.75	56.36	60.09	63.56
**South**											
0-19	1,026	865	871	874	900	1073	783	852	843	853	858
20-39	5,867	5,687	6,279	6,220	6,083	6,884	7,484	7,482	7,595	7,391	7029
40-59	5,050	5,082	5,289	5,316	4,997	5,020	5,221	5,471	5,860	6,673	7608
60 years+	1,864	1,729	1,773	1,907	2,116	2,373	2,355	2,458	2,587	2,767	2941
Total	13,807	13,363	14,212	14,317	14,096	15,350	15,844	16,262	16,886	17,684	18,435
CR/100 thousand	35.81	33.05	34.96	34.27	32.96	35.08	35.45	35.72	36.50	37.73	38.93
SR/100 thousand	37.99	34.21	33.73	32.38	30.67	32.51	32.78	33.10	34.04	35.35	36.48
**Center-West**											
0-19	490	420	324	388	454	358	330	365	349	348	346
20-39	1,983	2,076	2,175	2,740	2,851	3,267	3,441	3,428	3,367	3,199	3165
40-59	1,767	1,849	1,893	2,135	1,956	2,119	2,346	2,693	3,255	3,973	4466
60 years+	930	902	897	967	1,022	1,064	1,069	1,113	1,209	1,395	1644
Total	5,170	5,247	5,289	6,230	6,283	6,808	7,186	7,599	8,180	8,915	9621
CR/100 thousand	28.07	26.50	25.61	28.29	27.34	28.46	28.95	29.62	30.96	32.87	34.67
SR/100 thousand	33.77	30.82	26.98	28.41	26.80	27.23	27.18	27.42	28.35	29.77	31.02
**Brazil**											
0-19	10,705	9,511	8,801	9,056	8,805	9,881	9,020	10,011	10,712	10,795	10,907
20-39	51,583	50,630	52,329	52,994	55,106	65,260	73,416	77,330	79,191	78,961	80,360
40-59	43,255	41,841	41,775	41,138	39,260	40,679	46,582	52,870	61,756	72,499	80,815
60 years+	16,803	16,194	15,876	16,479	17,684	19,561	20,506	21,556	25,050	26,631	29,345
Total	122,346	118,176	118,781	119,667	120,855	135,381	149,524	161,766	176,709	188,886	201,426
CR/100 thousand	46.84	42.87	42.33	41.35	40.76	44.65	48.36	51.54	54.75	57.81	60.84
SR/100 thousand	52.43	46.83	42.90	40.92	39.46	42.56	45.50	48.15	51.04	53.84	56.59

**Women**											

**North**											
0-19	1,176	1,041	1,018	1,092	1,018	1,152	1,217	1,277	1,348	1,347	1358
20-39	3,615	3,364	3,372	3,167	3,070	3,215	3,829	4,305	4,699	5,038	5231
40-59	1,584	1,635	1,730	1,838	1,828	2,146	2,350	2,566	2,798	3,049	3420
60 years+	909	933	905	1056	1,106	1,415	1,578	1,830	2,061	2,277	2512
Total	7,284	6,973	7,025	7,153	7,022	7,928	8,973	9,978	10,906	11,711	12,520
CR/100 thousand	35.69	31.34	30.63	28.75	27.01	29.24	31.79	34.09	36.07	37.60	39.14
SR/100 thousand	43.90	38.17	34.07	31.48	28.77	30.48	32.12	33.74	35.14	36.17	37.26
**Northeast**											
0-19	3,244	2,785	2,273	2,115	1,840	1,868	1,651	1,527	1,602	1,552	1528
20-39	9,999	8,943	8,035	6,885	6,040	6,115	6,466	6,488	6,275	6,131	5955
40-59	5,761	5,570	5,116	4,872	4,620	4,692	4,727	4,861	5,039	5,364	5717
60 years+	3,373	3,199	3,052	2,968	2,796	3,083	3,309	3,543	3,797	3,977	4125
Total	22,377	20,497	18,476	16,840	15,296	15,758	16,153	16,419	16,713	17,024	17,325
CR/100 thousand	29.65	25.98	22.68	19.96	17.78	17.98	18.11	18.12	18.20	18.33	18.49
SR/100 thousand	32.74	28.28	23.15	19.79	17.22	17.10	16.97	16.79	16.73	16.79	16.90
**Southeast**											
0-19	3,889	3,350	3,201	3,274	3,124	3,224	3,057	3,124	3,348	3,370	3366
20-39	13,720	12,355	11,849	10,996	10,361	10,357	11,138	11,341	11,458	11,716	11,937
40-59	7,403	6,932	6,950	6,897	6,605	6,706	6,688	6,815	7,053	7,514	8027
60 years+	2,967	2,780	2,785	2,969	3,100	3,680	3,776	4,075	4,322	4,501	4639
Total	27,979	25,417	24,785	24,136	23,190	23,967	24,660	25,355	26,180	27,102	27,969
CR/100 thousand	24.22	20.84	20.01	18.76	17.58	17.76	17.89	18.05	18.34	18.74	19.14
SR/100 thousand	23.92	20.39	19.11	17.82	16.68	16.94	17.31	17.73	18.35	19.07	19.72
**South**											
0-19	869	732	774	722	699	726	621	614	642	650	654
20-39	3,526	3,250	3,245	3,033	2,852	2,752	2,932	2,927	2,914	2,885	2832
40-59	1,841	1,865	1,960	1,976	1,860	1,958	1,929	1,915	1,907	1,996	2148
60 years+	911	801	807	837	992	1,093	1,133	1,238	1,359	1,464	1528
Total	7,147	6,648	6,786	6,568	6,403	6,529	6,615	6,694	6,822	6,995	7161
CR/100 thousand	18.09	16.02	16.18	15.16	14.41	14.35	14.22	14.11	14.14	14.29	14.47
SR/100 thousand	18.17	15.86	15.45	14.32	13.49	13.47	13.46	13.49	13.68	14.00	14.29
**Center-West**											
0-19	416	350	302	314	342	278	247	224	180	178	175
20-39	1,217	1,167	998	1,085	1,056	946	925	905	890	849	788
40-59	676	719	758	825	783	769	755	750	782	833	895
60 years+	458	432	400	478	416	495	478	508	559	616	668
Total	2,767	2,668	2,458	2,702	2,597	2,488	2,404	2,386	2,412	2,477	2526
CR/100 thousand	14.93	13.34	11.70	12.10	11.13	10.22	9.50	9.11	8.92	8.91	8.86
SR/100 thousand	16.81	14.65	11.92	11.97	10.78	9.76	8.94	8.48	8.19	8.03	7.84
**Brazil**											
0-19	9594	8258	7568	7517	7023	7248	6771	6708	6925	6890	6866
20-39	32,077	29,079	27,499	25,166	23,379	23,385	25,219	25,873	26,158	26,399	26,366
40-59	17,265	16,721	16,514	16,408	15,696	16,271	16,459	16,888	17,532	18,715	20,173
60 years+	8,618	8,145	7,949	8,308	8,410	9,766	10,265	11,179	12,092	12,841	13,464
Total	67,554	62,203	59,530	57,399	54,508	56,670	58,712	60,648	62,707	64,845	66,868
CR/100 thousand	25.07	21.85	20.44	18.91	17.49	17.73	17.95	18.16	18.44	18.77	19.11
SR/100 thousand	25.97	22.34	19.95	18.18	16.62	16.77	16.97	17.23	17.60	18.03	18.45

CR = crude rate per 100,000; SR = standardized rate per 100,000.

Table 2 presents the number of deaths and crude and standardized mortality rates separated by sex. The highest standardized mortality rates were identified in the north and northeast in both sexes. The lowest rates were identified in the center-west for men and south for women.

**Table 2:** Number of cases and crude and standardized mortality rates per 100,000 inhabitants by sex in Brazil and macroregions in the observed (2002-2019) and projected (2020-2034) periods.

Region	Observed							Projected			
	02-04	05-07	08-10	11-13	14-16	17-19	20-22	23-25	26-28	29-31	32-34
**Men**											

**North**											
0-19	23	19	16	19	19	28	23	23	22	22	22
20-39	121	113	100	143	135	172	196	225	252	256	258
40-59	192	212	221	283	243	298	313	338	375	446	523
60 years+	260	299	318	371	360	401	433	468	495	520	567
Total	596	643	655	816	757	899	966	1,053	1,144	1,244	1,369
CR/100 thousand	2.85	2.82	2.80	3.22	2.87	3.28	3.39	3.58	3.78	4.00	4.30
SR/100 thousand	5.00	4.96	4.34	4.64	3.90	4.16	4.05	4.01	3.98	3.97	4.05
**Northeast**											
0-19	67	47	44	28	24	41	32	29	26	25	24
20-39	586	547	584	562	557	528	535	566	621	687	655
40-59	1,132	1,269	1,373	1,244	1,212	1,177	1,176	1,191	1,276	1,414	1,690
60 years+	1,181	1,253	1,246	1,169	1,163	1,201	1,186	1,230	1,304	1,422	1,561
Total	2,966	3,116	3,247	3,003	2,956	2,947	2,928	3,016	3,227	3,547	3,929
CR/100 thousand	4.09	4.10	4.15	3.74	3.63	3.57	3.49	3.56	3.77	4.11	4.52
SR/100 thousand	5.67	5.60	5.10	4.25	3.92	3.67	3.42	3.32	3.36	3.51	3.72
**Southeast**											
0-19	37	32	43	39	28	34	31	28	27	26	26
20-39	895	675	672	698	634	666	718	750	764	745	709
40-59	2,329	2,078	2,167	1,964	1,926	1,717	1,542	1,534	1,692	2,017	2,357
60 years+	1,662	1,491	1,533	1,467	1,555	1,520	1,500	1,495	1,497	1,533	1,601
Total	4,923	4,276	4,415	4,168	4,143	3,937	3,790	3,806	3,979	4,321	4,692
CR/100 thousand	4.45	3.66	3.75	3.42	3.31	3.07	2.89	2.85	2.93	3.14	3.37
SR/100 thousand	5.39	4.30	3.88	3.34	3.08	2.74	2.48	2.36	2.35	2.46	2.58
**South**											
0-19	12	8	3	7	9	6	7	7	7	7	7
20-39	193	181	181	135	163	176	219	249	271	268	263
40-59	496	426	407	404	399	427	480	529	590	710	838
60 years+	392	336	329	314	344	428	481	569	657	724	798
Total	1,093	951	920	860	915	1,037	1,186	1,354	1,525	1,709	1,905
CR/100 thousand	2.83	2.35	2.26	2.06	2.14	2.37	2.65	2.97	3.30	3.65	4.02
SR/100 thousand	3.45	2.72	2.31	1.98	1.96	2.07	2.23	2.41	2.58	2.77	2.98
**Center-West**											
0-19	14	7	8	5	7	7	7	7	7	7	7
20-39	67	64	61	71	71	86	87	82	76	73	70
40-59	180	161	178	185	183	200	226	261	311	364	409
60 years+	197	212	197	171	181	174	171	188	216	267	324
Total	458	444	444	432	442	467	490	538	609	710	810
CR/100 thousand	2.49	2.24	2.15	1.96	1.92	1.95	1.98	2.10	2.31	2.62	2.92
SR/100 thousand	3.79	3.28	2.67	2.25	2.06	1.95	1.88	1.89	1.99	2.16	2.31
**Brazil**											
0-19	153	113	114	98	87	116	102	96	93	91	90
20-39	1,862	1,580	1,598	1,609	1,560	1,628	1,759	1,876	1,986	2,045	1,987
40-59	4,329	4,146	4,346	4,080	3,963	3,819	3,806	3,910	4,237	4,797	5,527
60 years+	3,692	3,591	3,623	3,492	3,603	3,724	3,760	3,877	4,371	4,423	4,845
Total	10,036	9,430	9,681	9,279	9,213	9287	9,427	9,760	10,687	11,356	12,450
CR/100 thousand	3.84	3.42	3.45	3.21	3.11	3.06	3.05	3.11	3.31	3.48	3.76
SR/100 thousand	5.02	4.35	3.87	3.43	3.19	2.99	2.85	2.79	2.80	2.86	2.96

**Women**											

**North**											
0-19	17	13	23	18	19	17	18	17	17	17	17
20-39	64	67	72	55	55	80	63	63	66	67	68
40-59	80	72	96	97	105	124	144	158	166	170	159
60 years+	127	136	140	176	192	214	283	327	374	442	526
Total	288	288	331	346	371	435	507	565	623	695	770
CR/100 thousand	1.41	1.29	1.44	1.39	1.43	1.60	1.80	1.93	2.06	2.23	2.41
SR/100 thousand	2.38	2.15	2.04	1.92	1.87	1.93	2.06	2.07	2.06	2.07	2.06
**Northeast**											
0-19	49	43	29	24	18	20	17	15	14	14	13
20-39	286	268	248	193	188	164	157	140	130	130	122
40-59	436	416	410	338	351	332	283	270	266	259	275
60 years+	502	587	580	499	529	511	498	495	488	508	509
Total	1,273	1,314	1,267	1,054	1,086	1,027	954	921	898	910	919
CR/100 thousand	1.69	1.67	1.55	1.25	1.26	1.17	1.07	1.02	0.98	0.98	0.98
SR/100 thousand	2.09	2.00	1.69	1.26	1.21	1.06	0.92	0.82	0.75	0.72	0.69
**Southeast**											
0-19	47	41	35	36	33	22	25	24	24	24	24
20-39	384	318	300	268	259	229	236	232	229	214	201
40-59	498	506	454	440	402	383	330	317	326	369	411
60 years+	550	497	521	473	483	500	501	496	521	556	591
Total	1,479	1,362	1,310	1,217	1,177	1,134	1,091	1,068	1,100	1,163	1,227
CR/100 thousand	1.28	1.12	1.06	0.95	0.89	0.84	0.79	0.76	0.77	0.80	0.84
SR/100 thousand	1.36	1.15	0.98	0.84	0.75	0.68	0.62	0.58	0.57	0.58	0.59
**South**											
0-19	10	13	9	2	10	5	7	7	6	7	7
20-39	72	65	66	40	71	45	50	45	42	39	37
40-59	89	103	111	76	87	84	86	88	92	96	98
60 years+	138	131	128	90	126	133	128	141	157	184	217
Total	309	312	314	208	294	267	271	280	298	325	359
CR/100 thousand	0.78	0.75	0.75	0.48	0.66	0.59	0.58	0.59	0.62	0.66	0.73
SR/100 thousand	0.85	0.79	0.69	0.42	0.56	0.46	0.45	0.44	0.44	0.45	0.46
**Center-West**											
0-19	5	5	5	3	2	3	3	2	2	3	3
20-39	36	21	30	28	18	16	17	15	14	13	13
40-59	39	52	28	49	48	33	29	27	28	32	35
60 years+	85	74	71	71	45	48	43	43	47	52	55
Total	165	152	134	151	113	100	91	88	91	100	105
CR/100 thousand	0.89	0.76	0.64	0.68	0.48	0.41	0.36	0.34	0.34	0.36	0.37
SR/100 thousand	1.33	1.07	0.75	0.74	0.49	0.39	0.32	0.29	0.27	0.27	0.27
**Brazil**											
0-19	128	115	101	83	82	67	45	41	39	38	37
20-39	842	739	716	585	591	534	523	461	407	329	262
40-59	1,142	1,149	1,099	1,000	993	956	846	815	814	861	901
60 years+	1,402	1,425	1,440	1,309	1,375	1,406	1,406	1,413	1,455	1,544	1,616
Total	3,514	3,428	3,356	2,977	3,041	2,963	2,820	2,731	2,715	2,771	2,816
CR/100 thousand	1.30	1.20	1.15	0.98	0.98	0.93	0.86	0.82	0.80	0.80	0.80
SR/100 thousand	1.51	1.35	1.15	0.92	0.88	0.79	0.70	0.63	0.59	0.56	0.53

CR = crude rate per 100,000; SR = standardized rate per 100,000.

Table 3 reveals whether the changes between the last observed period and the last projected period were due to alterations in disease risk or changes in population size. In all macroregions and sexes, an increase in the number of new cases was projected, whereas a reduction in deaths was observed only in the northeast region and Brazil in women. The differences observed in new cases were attributed to an increased risk of contracting TB, whereas for deaths, they were due to changes in population size.

**Table 3:** Annual changes due to risk and population size in the incidence and mortality of pulmonary tuberculosis by sex and macroregion of the country in the last observed period (2017-2019) and projected period (2032-2034).

Sex Regions	Incidence				Mortality			
	N	Change (%)	Risk (%)	Population (%)	N	Change (%)	Risk (%)	Population (%)
**Men**								
North	14,887	96.8	70.2	26.6	470	52.3	−2.5	54.8
Northeast	14,645	41.8	28.6	13.2	982	33.3	−0.9	34.2
Southeast	29,765	47.4	39.9	7.5	755	19.2	−14.0	33.2
South	3,085	20.1	11.4	8.7	868	83.7	50.5	33.3
Center-west	2,813	41.3	19.6	21.7	343	73.4	27.7	45.7
Brazil	66,045	48.8	36.0	12.7	3,163	34.1	−6.0	40.0
**Women**								
North	4,592	57.9	26.4	31.5	335	76.9	12.3	64.6
Northeast	1,567	9.9	−4.3	14.2	−108	−10.5	−51.0	40.5
Southeast	4,002	16.7	10.4	6.3	93	8.2	−24.6	32.8
South	632	9.7	2.1	7.6	92	34.5	−3.1	37.6
Center-west	38	1.5	−25.0	26.5	5	5.0	−55.4	60.4
Brazil	10,198	18.0	6.3	11.7	−147	−5.0	−44.8	39.9

N = difference in absolute values of cases and deaths between the last projected period and the last observed period.

An increase in the number of cases was observed in both sexes and all macroregions, with the north region showing the highest increase in the risk of contracting the disease. In mortality, an increase was also identified, but it was attributed to changes in population size and a reduction in risk, except for the south and center-west in males and the north in females, which showed an increase in the risk of death.

Table 4 presents the results of the temporal trend across all periods. In the trend segments (TPC), a predominance of incidence reduction was observed in some regions and sexes until the 2014-2016 triennium, followed by growth until the last projected period. In mortality, the predominant trend is reduction, except in the center-west for males, where growth was observed from the 2017-2019 to 2032-2034 trienniums and in the south from the 2011-2013 to 2032-2034 trienniums. Most segments that include the projected period show stationary mortality. On the other hand, in the entire time series (Average 3-year Percent Change - ATPC), the reduction prevailed in both indicators, except for incidence in males, where growth was observed in the north, southeast, and Brazil.

**Table 4:** Temporal trend of standardized (per 100,000) incidence and mortality of pulmonary tuberculosis by sex and macroregion of the country in the observed (2002-2019) and projected (2020-2034) periods.

Characteristics	Segment		TPC	CI 95%		ATPC	CI 95%	
				Upper	Lower		Upper	Lower
**Incidence**								

**Men**								
North	2002-2004	2011-2013	−3.1^a^	−6.2	−1.5	1.1^a^	0.8	1.5
	2011-2013	2032-2034	3.0^a^	2.5	3.6			
Northeast	2002-2004	2014-2016	−3.6^a^	−4.0	−3.1	−0.4^a^	−0.6	−0.2
	2014-2016	2032-2034	1.7^a^	1.4	2.0			
Southeast	2002-2004	2011-2013	−2.8^a^	−3.1	−2.4	0.6^a^	0.5	0.7
	2011-2013	2032-2034	2.1^a^	2.0	2.3			
South	2002-2004	2014-2016	−1.4^a^	−2.0	−1.0	−0.1	−0.2	0.1
	2014-2016	2032-2034	0.9^a^	0.7	1.2			
Center-west	2002-2004	2008-2010	−3.9^a^	−5.2	−2.0	−0.4^a^	−0.7	−0.1
	2008-2010	2032-2034	0.5^a^	0.1	1.0			
Brazil	2002-2004	2011-2013	−3.2^a^	−3.7	−2.7	0.3^a^	0.1	0.4
	2011-2013	2032-2034	1.8^a^	1.6	2.0			
**Women**								
North	2002-2004	2014-2016	−3.3^a^	−4.1	−2.7	−0.4^a^	−0.6	−0.2
	2014-2016	2032-2034	1.5^a^	1.1	1.9			
Northeast	2002-2004	2014-2016	−5.3^a^	−5.6	−5.0	−2.2^a^	−2.3	−2.1
	2014-2016	2032-2034	−0.1	−0.3	0.1			
Southeast	2002-2004	2014-2016	−2.8^a^	−3.1	−2.4	−0.5^a^	−0.7	−0.4
	2014-2016	2032-2034	1.0^a^	0.8	1.2			
South	2002-2004	2014-2016	−2.3^a^	−2.9	−1.9	−0.8^a^	−0.9	−0.6
	2014-2016	2032-2034	0.3^a^	0.1	0.6			
Center-west	2002-2004	2020-2022	−3.3^a^	−4.9	−2.7	−2.4^a^	−2.7	−2.1
	2020-2022	2032-2034	−1.0	−2.1	1.3			
Brazil	2002-2004	2014-2016	−3.6^a^	−3.9	−3.3	−1.1^a^	−1.2	−1.0
	2014-2016	2032-2034	0.7^a^	0.5	0.8			

**Mortality**								

**Men**								
North	2002-2004	2014-2016	−1.8^a^	−2.4	−1.5	−0.8^a^	−1.0	−0.7
	2014-2016	2032-2034	−0.2	−0.4	0.2			
Northeast	2002-2004	2020-2022	−3.2^a^	−6.0	−2.3	−1.6^a^	−2.2	−1.1
	2020-2022	2032-2034	0.8	−1.0	4.8			
Southeast	2002-2004	2020-2022	−4.1^a^	−6.4	−3.2	−2.3^a^	−2.9	−1.9
	2020-2022	2032-2034	0.5	−1.4	4.7			
South	2002-2004	2011-2013	−6.4^a^	−7.3	−5.3	−0.5^a^	−0.7	−0.2
	2011-2013	2032-2034	2.1^a^	1.7	2.5			
Center-west	2002-2004	2017-2019	−4.7^a^	−6.5	−3.5	−1.6^a^	−2.1	−1.1
	2017-2019	2032-2034	1.5^a^	0.3	3.5			
Brazil	2002-2004	2017-2019	−3.5^a^	−4.7	−2.8	−1.7^a^	−2.0	−1.4
	2017-2019	2032-2034	0.1	−0.6	1.4			
**Women**								
North	2002-2004	2011-2013	−2.3^a^	−4.4	−0.7	−0.4	−0.7	0.1
	2011-2013	2032-2034	0.5	0.0	2.2			
Northeast	2002-2004	2023-2025	−4.6^a^	−6.0	−4.1	−3.7^a^	−4.2	−3.4
	2023-2025	2032-2034	−1.6	−3.5	0.9			
Southeast	2002-2004	2020-2022	−4.4^a^	−5.6	−3.7	−2.7^a^	−3.1	−2.3
	2020-2022	2032-2034	−0.1	−1.4	2.7			
South	2002-2004	2011-2013	−6.9^a^	−12.1	−3.2	−2.5^a^	−3.2	−1.6
	2011-2013	2032-2034	−0.6	−1.7	2.1			
Center-west	2002-2004	2020-2022	−7.8^a^	−8.8	−7.2	−5.4^a^	−5.8	−5.0
	2020-2022	2032-2034	−1.5	−3.0	0.9			
Brazil	2002-2004	2011-2013	−5.5^a^	−7.8	−3.5	−3.6^a^	−4.0	−3.1
	2011-2013	2032-2034	−2.8^a^	−3.6	−0.1			

ATPC, average 3-year percent change; CI 95%, 95% confidence interval, upper and lower; TPC, 3-year percent change.

a Statistically significant result *P* <0.05.

## Discussion

This research presented relevant evidence regarding the future of pulmonary TB in Brazil. The projections are not favorable to the globally established targets. The highest incidence and mortality rates were found in the north and northeast regions of Brazil. Between 2017-2019 and 2032-2034, more new cases and deaths will occur in both sexes. However, this was attributed to changes in population size. The projected trend is a reduction in standardized incidence and mortality, especially in women.

The main limitation of this study relates to the use of secondary data owing to the presence of underreporting, data incompleteness, and reporting errors. In addition, this research did not differentiate between other types of TB, which have distinct clinical and epidemiologic implications, and did not consider external factors that could influence the projections.

Despite the limitations, the findings of this research are of great epidemiologic and operational value due to the long-term forecasting and differentiation of multiple sociodemographic aspects, such as sex, age group, and major regions of the country. The projected period extends beyond the maximum deadline set by the WHO for the eradication of TB (until 2035), according to the strategy adopted in 2015, and aligns with the goals of the Sustainable Development Goals (2030) [4]. In addition to the national representation of this research, the design of this study is unprecedented in the context of TB because no similar studies were found. Therefore, it is recommended that public policies be reviewed and adapted, focusing on the implementation of strategies that can further optimize the effectiveness of TB prevention and control programs to ensure success in achieving the targets. The results obtained provide a solid foundation for revising national, regional, and local public policies. The implementation of more effective TB prevention and control strategies is crucial to avoid future scenarios that could compromise the established goals. This study can serve as a guide for the development of targeted interventions, optimizing public health programs and strengthening the health system's capacity to respond to the specific demands of different regions of the country.

In this research, incidence and mortality rates demonstrate the disparities between the macroregions of the country, with higher rates occurring predominantly in the north and northeast. The differences in pulmonary TB indicators among the major regions of the country are not new. Zille *et al.* [17], in their study on the correlation with socioeconomic factors, identified that higher income levels and educational attainment and lower economic inequality, were associated with lower incidence and mortality rates of pulmonary TB. This scenario has also been observed worldwide, where countries with lower Human Development Indices, especially in Africa and Latin America, presented higher incidence rates of the disease [18]. Thus, it is known that the north and northeast regions of Brazil face social, economic, and health disadvantages. These aspects are determinants of the disease burden and contribute to unfavorable outcomes, such as treatment abandonment and death.

The incidence and mortality of pulmonary TB were higher in males, whereas females were the only group that showed projections of meeting the targets. The predominance among men is expected and well-documented in the literature [19,20]. According to a systematic review with meta-analysis, the duration of infection in males is longer than in females, thus increasing the likelihood of generating new secondary infections [21]. In addition, men are responsible for transmitting the disease to men, women, and children [22]. Recommendations for addressing TB worldwide can no longer overlook gender inequalities because men tend to bear a heavier burden of the disease and have less access to diagnosis and treatment. Therefore, the impact of TB on males should be consistently considered in prevention and treatment policies to achieve global targets.

The standardized incidence rate of pulmonary TB showed an overall reduction in both sexes and in most macroregions. Since 1990 (up to 2010), the incidence of TB in Brazil had an annual reduction of 3.2% (95% CI = −3.3 to −3.2, *P* <0.001) per year [23]. More recent data from the period 2006-2017 demonstrated a similar pattern of −1.7% (95% CI = −2.0 to −1.4, *P* <0.001) per year [24]. Thus, the results presented in this study are consistent with the literature. Over the past few decades, numerous significant changes have occurred in national TB policies that have contributed to the current scenario, such as the launch of the Strategic Plan for TB Control in Brazil in 2006, the change in the TB treatment regimen in 2009, and the National Plan to End TB in 2017 [3]. Since then, the Ministry of Health has intensified its activities to control the disease in light of the goals established by the WHO for 2035, where less than 55 cases per 100,000 are expected by 2025 (<50%), less than 20 per 100,000 by 2030 (<80%), and less than 10 cases per 100,000 by 2035 (<90%) [25]. Despite the favorable scenario, the rates are still far from the proposed targets. Brazil has the appropriate size to reduce the number of new TB cases [2] due to free access to diagnosis and treatment of the disease, as well as a monitoring and follow-up care network. However, the coordination of care networks and increased public investment should be a priority, in addition to greater efforts by the population and their leaders in the preventive fight against the disease.

The standardized mortality rates showed a general downward trend across all macroregions. The literature clearly indicates a reduction in TB mortality in Brazil in recent years [26,27], a fact also evidenced in this research. The reduction in incidence [28] and treatment abandonment [29] over the past decades, along with various strategies to combat the disease in the country—such as strengthening actions in primary care, active and passive surveillance, active case finding of respiratory symptoms, universal access to treatment [3], among others—have contributed to reducing TB deaths. Even so, many deaths may occur, especially in disadvantaged areas such as the north and northeast. Therefore, intensifying existing strategies with a focus on high-risk areas can further improve this scenario. It is important to emphasize that expanding efforts by public authorities, managers, and professionals is crucial to overcoming the barriers that hinder the achievement of better.

In this study, the increase in new cases and deaths in the last projected period observed in the north region was predominantly explained by an increased risk of contracting and dying of TB. Despite the lack of studies with similar results for comparison, a study in indigenous populations showed that residing in the north increases the chance of death by 2.8 times (odds ratio = 2.8; 95% CI = 1.1-7.1) [30]. A higher risk of contracting or dying from TB in the north can be explained by multiple factors. The highest rates and clusters of risk for TB are found in this region [24,27], which is the socioeconomically least developed, presenting the most unfavorable indicators, such as gross domestic product per capita, Human Development Index, and Gini index [24,31]. There is a negative correlation between Human Development Index and Gini index, suggesting that the lower the Human Development Index and the higher the economic inequality, the worse the TB indicators (incidence, cure, treatment abandonment, and recurrence) [17]. The low development status in the north region directly impacts the provision of actions and services for prevention, health promotion, surveillance, and care for people with TB. It also affects the coverage of primary health care and the annual average of TB hospitalizations because the region has one of the lowest coverage rates in the country [31]. Therefore, addressing this challenging scenario requires a multifaceted approach. Strengthening policies to reduce inequalities and manage TB in these regions is imperative. Expanding programs, financial investment by governments, investment in research, and integrating emerging technologies, such as telemedicine, can help change the regional scenario.

## Conclusion

In summary, the highest standardized incidence and mortality rates were observed in men, especially in the north and northeast regions. The difference in the number of new cases and deaths between the last observed period and the last projected period showed an increase in both cases and deaths. The differences in cases were attributed to a higher risk of illness, whereas in deaths, they were due to population growth. The trend in standardized rates predominantly showed a reduction in incidence and mortality in both sexes by 2034.

## Declarations of competing interest

The authors have no competing interests to declare.

## References

[1] World Health Organization. Global tuberculosis report, https://www.who.int/publications-detail-redirect/9789241565394; 2016 [accessed 25 April 2024].

[2] Barreira D. (2018). Os desafios para a eliminação da tuberculose no Brasil. Epidemiol Serv Saúde.

[3] Ministério da Saúde, Brasil (2021).

[4] Brasil NU. Sobre o nosso trabalho para alcançar os Objetivos de Desenvolvimento Sustentável no Brasil. Objetivos de Desenvolvimento Sustentável, https://brasil.un.org/pt-br/sdgs; 2023 [accessed 17 July 2023].

[5] Benchimol EI, Smeeth L, Guttmann A, Harron K, Moher D, Petersen I (2015). The REporting of studies Conducted using Observational Routinely-collected health Data (RECORD) Statement. PLoS Med.

[6] National Cancer Institute. SEER data & software. Standard populations (millions) for age-adjustment - SEER population datasets. SEER 2024, https://seer.cancer.gov/stdpopulations/index.html; 2024 [accessed 22 April 2024].

[7] Instituto Brasileiro de Geografia e Estatística. Censo, 2010, https://censo2010.ibge.gov.br/; 2020 [accessed 20 August 2021].

[8] Instituto Brasileiro de Geografia e Estatística. Censo de 2022, https://www.ibge.gov.br/estatisticas/sociais/trabalho/22827-censo-demografico-2022.html,; 2023 [accessed 29 January 2024].

[9] Instituto Brasileiro de Geografia e Estatística (2013).

[10] Berra TZ, Ramos ACV, Alves YM, Tavares RBV, Tartaro AF, do Nascimento MCD (2022). Impact of COVID-19 on tuberculosis indicators in Brazil: a time series and spatial analysis study. Trop Med Infect Dis.

[11] Rutherford MJ, Lambert PC, modeling Thompson JR.Age–period–cohort (2010). STATA J.

[12] Chen J, Qiu Y, Wu W, Yang R, Li L, Yang Y (2023). Trends and projection of the incidence of active pulmonary tuberculosis in Southwestern China: age-period-cohort analysis. JMIR Public Health Surveill.

[13] Ota M, Hirao S, Uchimura K. (2023). Age-period-cohort analysis on tuberculosis cases in Japan, 1953–2022. Int J Mycobacteriology.

[14] Møller B, Fekjaer H, Hakulinen T, Sigvaldason H, Storm HH, Talbäck M (2003). Prediction of cancer incidence in the Nordic countries: empirical comparison of different approaches. Stat Med.

[15] Santos CAD, Souza DLB. (2019). Melanoma mortality in Brazil: trends and projections, 1998–2032. Ciênc Saúde Coletiva.

[16] Surveillance Research Program. Empirical quantile confidence interval. Joinpoint help system, https://surveillance.cancer.gov/help/joinpoint/setting-parameters/method-and-parameters-tab/apc-aapc-tau-confidence-intervals/empirical-quantile; 2023 [accessed 28 August 2023].

[17] Zille AI, Werneck GL, Luiz RR, Conde MB. (2019). Social determinants of pulmonary tuberculosis in Brazil: an ecological study. BMC Pulm Med.

[18] Castañeda-Hernández DM, Tobón-García D, Rodríguez-Morales AJ (2013). [Association between tuberculosis incidence and the Human Development Index in 165 countries of the world]. Rev Peru Med Exp Salud Publica.

[19] Pereira A, Hillesheim D, da Silva FM, Valim RCS, Hallal ALC (2022). Série histórica da taxa de incidência de tuberculose em Santa Catarina: análise de uma década, 2010–2019. Epidemiol e Serviços Saúde.

[20] Cecilio HPM, Santos Ade L, Marcon SS, Latorre M do RD de O, Mathias TA de F, Rossi RM (2018). Tendência da mortalidade por tuberculose no estado do Paraná, Brasil, 1998–2012. Ciênc Saúde Coletiva.

[21] Horton KC, MacPherson P, Houben RMGJ, White RG, Corbett EL. (2016). Sex differences in tuberculosis burden and notifications in low- and middle-income countries: a systematic review and meta-analysis. PLoS Med.

[22] Dodd PJ, Looker C, Plumb ID, Bond V, Schaap A, Shanaube K (2016). Age- and sex-specific social contact patterns and incidence of Mycobacterium tuberculosis infection. Am J Epidemiol.

[23] Guimarães RM, Lobo Ade P, Siqueira EA, Borges TFF, Melo SCC (2012). Tuberculose, HIV e pobreza: tendência temporal no Brasil, Américas e mundo. J Bras Pneumol.

[24] Melo MC de, Barros H, Donalisio MR (2020). Tendência temporal da tuberculose no Brasil. Cad Saúde Publ.

[25] World Health Organization. Global tuberculosis report, https://www.who.int/publications-detail-redirect/9789240083851; 2023 [accessed 22 May 2024].

[26] Queiroz JR de, Vieira NF, Oliveira MD da S, Maia LG, Figueiredo RC de, Gonzalez RIC (2024). Tendência da mortalidade por tuberculose e relação com o índice sóciodemográfico no Brasil entre 2005-2019. Ciênc Saúde Coletiva.

[27] Souza CDF de, Paiva JPS de, Silva LF da, Leal TC, Magalhães M de.AFM (2019). Tendência da mortalidade por tuberculose no Brasil, 1990–2015: análise por pontos de inflexão. J Bras Pneumol.

[28] Paiva JPS de, Magalhães MAFM, Leal TC, Silva LF da, Silva LG da, Carmo RF do (2022). Time trend, social vulnerability, and identification of risk areas for tuberculosis in Brazil: an ecological study. PLoS One.

[29] Soeiro VMda S, Caldas A de JM, Ferreira TF (2022). Abandono do tratamento da tuberculose no Brasil, 2012–2018: tendência e distribuição espaço-temporal. Ciênc Saúde Coletiva.

[30] Viana PVde S, Codenotti SB, Bierrenbach AL, Basta PC. (2019). Tuberculose entre crianças e adolescentes indígenas no Brasil: fatores associados ao óbito e ao abandono do tratamento. Cad Saúde Publ.

[31] Cortez AO, Melo AC, Neves LO, Resende KA, Camargos P. (2021). Tuberculosis in Brazil: one country, multiple realities. J Bras Pneumol.

